# Challenges in Clinicogenetic Correlations: One Phenotype – Many Genes

**DOI:** 10.1002/mdc3.13163

**Published:** 2021-03-02

**Authors:** Rahul Gannamani, Sterre van der Veen, Martje van Egmond, Tom J. de Koning, Marina A.J. Tijssen

**Affiliations:** ^1^ Department of Neurology University of Groningen, University Medical Centre Groningen Groningen The Netherlands; ^2^ Department of Genetics University of Groningen, University Medical Centre Groningen Groningen The Netherlands; ^3^ Expertise Centre Movement Disorders Groningen University Medical Centre Groningen Groningen The Netherlands; ^4^ Pediatrics, Department of Clinical Sciences Lund University Lund Sweden

**Keywords:** movement disorder, phenotype, genotype, neurogenetics, genetics

## Abstract

**Background:**

In the field of movement disorders, what you see (phenotype) is seldom what you get (genotype). Whereas 1 phenotype was previously associated to 1 gene, the advent of next‐generation sequencing (NGS) has facilitated an exponential increase in disease‐causing genes and genotype–phenotype correlations, and the “one‐phenotype‐many‐genes” paradigm has become prominent.

**Objectives:**

To highlight the “one‐phenotype‐many‐genes” paradigm by discussing the main challenges, perspectives on how to address them, and future directions.

**Methods:**

We performed a scoping review of the various aspects involved in identifying the underlying molecular cause of a movement disorder phenotype.

**Results:**

The notable challenges are (1) the lack of gold standards, overlap in clinical spectrum of different movement disorders, and variability in the interpretation of classification systems; (2) selecting which patients benefit from genetic tests and the choice of genetic testing; (3) problems in the variant interpretation guidelines; (4) the filtering of variants associated with disease; and (5) the lack of standardized, complete, and up‐to‐date gene lists. Perspectives to address these include (1) deep phenotyping and genotype–phenotype integration, (2) adherence to phenotype‐specific diagnostic algorithms, (3) implementation of current and complementary bioinformatic tools, (4) a clinical‐molecular diagnosis through close collaboration between clinicians and genetic laboratories, and (5) ongoing curation of gene lists and periodic reanalysis of genetic sequencing data.

**Conclusions:**

Despite the rapidly emerging possibilities of NGS, there are still many steps to take to improve the genetic diagnostic yield. Future directions, including post‐NGS phenotyping and cohort analyses enriched by genotype–phenotype integration and gene networks, ought to be pursued to accelerate identification of disease‐causing genes and further improve our understanding of disease biology.

In the field of movement disorders, what you see (phenotype) is seldom what you get (genotype). Whereas 1 phenotype was previously associated with 1 gene, for example, myoclonus‐dystonia with *SGCE* or benign hereditary chorea with *NKX2‐1*, advances in sequencing techniques, usually referred to as next‐generation sequencing (NGS), have revolutionized DNA diagnostics and facilitated an exponential increase in disease‐causing genes and genotype–phenotype correlations.^1^, ^2^ With several hundred genes now implicated in movement disorder pathophysiology, including a number of genes that are yet to be replicated, it becomes more and more difficult to identify the 1 disease‐causing gene among the many possible genetic defects.^3^ Illustrative examples include dystonia, recessive cerebellar ataxia, and myoclonus syndromes, with each associated with more than 100 genes.^4^, ^5^, ^6^ This is what we call the “one‐phenotype‐many‐genes” paradigm.

Although movement disorders can consist solely of 1 movement disorder—isolated dystonia for instance—they typically consist of a combination of 1 or more movement disorders and associated features.^7^ Examples include myoclonus‐dystonia, genetic syndromes with movement disorders and epilepsy, and neurodegeneration with brain iron accumulation.^7^, ^8^, ^9^ The labels that are used to differentiate isolated movement disorders include, but are not limited to, ataxia, chorea, dystonia, myoclonus, parkinsonism, and tremor.^10^ In this review, we use the term *phenotype* broadly: incorporating movement disorders consisting of only 1 movement disorder (ie, isolated syndromes) as well as those consisting of multiple movement disorders (ie, combined syndromes).

A systematic approach to the evaluation of a patient with a movement disorder, including a careful history and observation of the movement disorder and associated features, is the critical starting point to the diagnostic work‐up.^10^ Once the phenotype is established, the differential diagnostic process seeking to identify the underlying cause follows.^10^ First, acquired and treatable causes need to be quickly sought for and ruled out with a range of clinical investigations such as blood and cerebrospinal fluid samples and neuroimaging.^1^, ^4^, ^5^, ^6^, ^8^, ^9^, ^11^, ^12^, ^13^, ^14^, ^15^, ^16^, ^17^, ^18^ Ultimately, genetic testing is used to determine the underlying molecular cause if 1 is suspected.

Unfortunately, genetic testing is not yet readily available in a significant number of countries. Furthermore, if available, the diagnostic yield of movement disorders ranges from only 10.1% to 61.8%, with yield varying depending on the phenotype, population, mode of inheritance, and chosen testing methodology.^19^, ^20^, ^21^ The large number of undiagnosed patients with a genetically suspected movement disorder suggests that there are still several, yet to be discovered, movement disorder genes and phenotype–genotype relationships (also in already known genes). Limitations in testing methodologies such as short read lengths and inadequate sequencing of the region of interest or a lack of parental data for trio analysis and differentiation of de novo and compound heterozygous variants are contributing factors.^22^, ^23^

There are 4 noteworthy biological phenomena that pose challenges in clinicogenetic correlations. These are phenocopy, phenotypic pleiotropy, polygenic heritance, and the focus of this review: genetic heterogeneity. A phenocopy is defined as an environmentally induced, nonhereditary phenotype of 1 individual that is highly comparable to the genetically determined phenotype of another individual.^24^ In other words, the phenocopy induced by nongenetic factors mimics the phenotype caused by a genetic defect. Phenotypic pleiotropy is the notion that 1 genetic defect can cause 2 or more seemingly unrelated phenotypic effects (disorders) in different individuals, as illustrated by the presence of the same genes in multiple movement disorder diagnostic panels. *ADCY5* is a characteristic example, with *ADCY5*‐related disorders presenting with a variety of phenotypes, including paroxysmal chorea, myoclonus, and dystonia.^25^ Polygenic inheritance is where variants in multiple genes contribute to the risk of a disorder.^3^ This is likely the case in more common phenotypes such as parkinsonism and essential tremor.^26^, ^27^ Genetic heterogeneity can be referred to more simply as the “one‐phenotype‐many‐genes” paradigm. It refers to the crux that 1 distinct phenotype can be the result of different genetic mechanisms, that is, mutations in different single genes can lead to the same downstream effects and phenotype in different individuals.^28^

The aim of this review is to highlight the one‐phenotype‐many‐genes paradigm by discussing the challenges of identifying the 1 disease‐causing gene among the many possible genetic defects that may be responsible for a certain movement disorder phenotype, perspectives on how to address them, and future directions to improve the diagnostic yield. We use dystonia as an illustrative example throughout the various sections.

## Classification of the Movement Disorder Phenotype

### Challenge 1: The Lack of Gold Standards, Overlap in Clinical Spectrum of Different Movement Disorders, and Variability in the Interpretation of Classification Systems

Phenotyping guides the diagnostic work‐up of movement disorders. As such, inaccurate classification leads to poor specification of molecular diagnostic testing and likely to a low diagnostic yield. A broad challenge within the field of movement disorders is the lack of a gold standard for many phenotypes. For example, dystonia is a phenotype defined as *sustained or intermittent muscle contractions causing abnormal*, *often repetitive*, *movements*, *postures*, *or both*.^29^ Nevertheless, because of the lack of a diagnostic gold standard, recognizing and characterizing dystonia appropriately heavily relies on a clinician's experience and intuition.^30^ Unfortunately, distinction can be difficult as overlap exists at the outer edges of the clinical spectrum of different movement disorders (eg, jerky movements in myoclonus‐dystonia can mimic jerky movements of benign hereditary chorea), and patients can suffer from multiple movement disorders at the same time. In addition, factors such as abnormal motor development in children and fluctuations in symptom expression may lead clinicians to interpret movement disorder classification systems differently.^30^ Consequently, consistently and accurately classifying the movement disorder phenotype is a major problem.^31^

### Perspective 1: Deep Phenotyping and Genotype–Phenotype Integration

In neurology, phenotyping comes from a medical history, anamnestic interview including characterization of nonmotor symptoms, physical examination, electrophysiology and neuroimaging, metabolite sampling from blood and cerebrospinal fluid, and more recently, wearable sensors and other smart devices. Deep phenotyping “shows the different dimensions of a disease,” and in a 2015 *Nature* article, it was described as a process that “gathers details about disease manifestations in a more individual and finer‐grained way, and uses sophisticated algorithms to integrate the resulting wealth of data with other kinds of information.”^32^, ^33^

Although the depth of a phenotype is a function of the number of areas evaluated, the quality of the assessment of each domain, and the duration of observation, a number of critical questions remain unanswered.^34^ When is an individual considered “deeply phenotyped”? To what extent does such an operational definition differ per phenotype? Will this definition need to be dynamically revised as new biomarkers are discovered? Consensus among clinicians and centers with complementary domain‐specific expertise is the first step toward efficient, consistent, and sufficient characterization of the movement disorder phenotype. Second, making sense of the resulting wealth of data beckons the use of machine learning and artificial intelligence; rapidly developing approaches that may become an important aid in the diagnosis, prognosis, and management of neurological disorders.^35^ A number of strides have already been made, particularly in the field of neuroimaging, although computational models are only as powerful as the data they rely on, and there is work yet to be done.^36^

Whereas gene mutation databases have typically been labeled with limited and sparse phenotype information, initiatives such as MDSGene are beginning to turn the tide in the field of movement disorders.^37^ MDSGene encompasses genetic, phenotypic, and clinical data extracted through systematic screens of relevant literature and standardized extraction protocols. As initiatives integrating genotype and phenotype data continue to accelerate, we can look forward to new biomarkers, genotype–phenotype association strengthening, further discovery of new disease‐causing genes, and an increased understanding of disease biology for more targeted therapies.

## The (Molecular) Diagnostic Work‐Up of a Movement Disorder Patient

### Challenge 2: Selecting Which Patients Benefit from Genetic Tests and the Choice of Genetic Testing

The objective of the diagnostic work‐up is to identify the underlying cause and guide the treatment strategy. As movement disorders can be acquired or inherited, ruling out acquired causes and deciding which patients do not need to be subjected to genetic testing is an important step. Hereafter, when a genetic cause is suspected, the challenge is to choose which genetic test is appropriate, for instance: single‐gene testing (Sanger sequencing), array‐comparative genomic hybridization, single nucleotide polymorphism array, polymerase chain reaction (PCR), Southern blot, multiplex ligation‐dependent probe amplification, or NGS. When NGS is the preferred technique, diagnostic platforms include: targeted resequencing (TRS) of multiple disease‐associated genes in a panel, whole‐exome sequencing (WES), whole‐genome sequencing (WGS), and mitochondrial genome (mtDNA) analysis. Moreover, recent advancements in long‐read (LR) sequencing technologies—read lengths of >10 kilobases from single DNA molecules, free from any PCR‐related bias—have resulted in the discovery of a number of new disease‐causing mutations and added another dimension of complexity by highlighting limitations of conventional (short‐read) NGS, including WES and even WGS.^22^, ^38^, ^39^

Although patients can present with a highly characteristic combination of signs and symptoms that are strongly suggestive of a specific genetic syndrome or disorder, the combination of overlap in clinical spectrum of different movement disorders and genetic heterogeneity makes a priori prediction of the mutated gene prone to errors—even for highly specialized movement disorders experts.^40^ Consequently, in patients presenting with a genetically determined or suspected movement disorder such as dystonia, where many potential candidate genes can explain the phenotype, methods that capture many genes (eg, TRS and WES) have been the prevailing choices of the past decade.^41^, ^42^, ^43^, ^44^ However, this is not to say that these methods are always the appropriate choice, nor that they will continue to prevail going forward. Illustrative examples where other methods such as Southern blot, triplet repeat primed PCR, mtDNA, or LR‐WGS analysis are more appropriate include the following: CAG trinucleotide repeat expansion in *HTT* (Huntington's disease), GAA trinucleotide repeat expansion in *FXN* (Friedrich's ataxia), mitochondrial defects (myoclonus epilepsy with ragged red fibers), intronic pentanucleotide TTTCA and TTTTA repeat insertions (familial cortical myoclonic tremor with epilepsy), and short interspersed nuclear element (SINE)‐variable number of tandem repeat (VNTR)‐*Alu* (SVA) retrotransposon insertion in *TAF1* (X‐linked dystonia parkinsonism).^45^, ^46^, ^47^, ^48^, ^49^ In sum, the selection of genetic testing is highly context dependent, and it is advisable to adhere to the latest phenotype‐specific diagnostic algorithms.

### Perspective 2: Adherence to Phenotype‐Specific Diagnostic Algorithms

As acquired and genetically determined movement disorders have their own nuances, a number of diagnostic algorithms have been curated to help guide clinicians through the diagnostic work‐up of a specific movement disorder. Table 1 provides an overview of the most recently published proposed diagnostic approaches for a range of movement disorder phenotypes and can help clinicians to select those patients that may benefit from genetic testing. Patients with a relatively high a priori likelihood for a monogenetic disorder are those with a pediatric onset, complex phenotype, positive family history, indications from laboratory or neuroradiological testing, and/or no verified diagnosis after the diagnostic work‐up.^21^

**TABLE 1:** *Overview of most recently published proposed diagnostic approaches in the field of movement disorders to guide clinicians in the identification of the underlying etiology*

Phenotype	Number of Genes Implicated	Reference for Diagnostic Approach
Parkinson's disease	19 risk factors	Payne K, Walls B, Wojcieszek J. Approach to assessment of Parkinson disease with emphasis on genetic testing. Med Clin North Am 2019^15^
Tremor	13 genes	van de Wardt J, van der Stouwe AMM, Dirkx M. Systematic clinical approach for diagnosing upper limb tremor. J Neurol Neurosurg Psychiatry 2020^16^
Dominant cerebellar ataxia	40 genes	de Silva RN, Vallortigara J, Greenfield J, Hunt B, Giunti P, Hadjivassiliou M. Diagnosis and management of progressive ataxia in adults. Pract Neurol 2019^12^
Recessive cerebellar ataxia	117 genes	Beaudin M, Matilla‐Dueñas A, Soong BW, et al. The classification of autosomal recessive cerebellar ataxias: a consensus statement from the Society for Research on the Cerebellum and Ataxias Task Force. Cerebellum 2019^5^
Dystonia	147 genes	van Egmond ME, Kuiper A, Eggink H, et al. Dystonia in children and adolescents: A systematic review and a new diagnostic algorithm. J Neurol Neurosurg Psychiatry 2015^4^
Myoclonus	116 genes	Zutt R, van Egmond ME, Elting JW, et al. A novel diagnostic approach to patients with myoclonus. Nat Rev Neurol 2015^6^
Chorea	20 genes	Termsarasab P. Chorea. Continuum 2019^14^
Myoclonus‐dystonia	14 genes	Menozzi E, Balint B, Latorre A, et al. Twenty years on: Myoclonus‐dystonia and ε‐sarcoglycan—Neurodevelopment, channel, and signaling dysfunction. Mov Disord 2019^1^
Dystonia‐ataxia	74 genes	Rossi M, Balint B, Millar Vernetti P, Bhatia KP, Merello M. Genetic dystonia‐ataxia syndromes: Clinical Spectrum, diagnostic approach, and treatment options. Mov Disord Clin Pract 2018^18^
Hereditary spastic paraplegia	67 genes	Shribman S, Reid E, Crosby AH, Houlden H, Warner TT. Hereditary spastic paraplegia: From diagnosis to emerging therapeutic approaches. Lancet Neurol 2019^11^
Paroxysmal movement disorders and episodic ataxia	26 genes	Garone G, Capuano A, Travaglini L. Clinical and genetic overview of paroxysmal movement disorders and episodic ataxias. Int J Mol Sci 2020^13^
Genetic epilepsy‐dyskinesia spectrum	73 genes	Papandreou A, Danti FR, Spaull R, Leuzzi V, Mctague A, Kurian MA. The expanding spectrum of movement disorders in genetic epilepsies 2020^8^
Neurodegeneration with brain iron accumulation	10 genes	Salomão RPA, Pedroso JL, Gama MTD, et al. A diagnostic approach for neurodegeneration with brain iron accumulation: Clinical features, genetics and brain imaging. Arq Neuropsiquiatr 2016^9^
Primary familial brain calcification	4 genes	Quintáns B, Oliveira J, Sobrido MJ. Primary familial brain calcifications. *Handbook of Clinical Neurology* 2018^17^

The proposed diagnostic approaches are accompanied with (extensive) lists of genes associated with each phenotype, underscoring the one‐phenotype‐many‐genes paradigm. Moreover, there is partial overlap of the genes in the various lists. Genes being represented in multiple lists exemplify the notion of phenotypic pleiotropy, so 1 genetic defect can cause 2 or more seemingly unrelated movement disorders in different individuals. Conversely, substantial differences between the gene lists reinforce the importance of accurate phenotyping for determining a molecular diagnosis.

### Challenge 3: Problems in the Variant Interpretation Guidelines

Finding a previously described mutation in a known disease‐causing gene in a patient with a compatible phenotype is clear cut. However, because of the lack of robust phenotype–genotype associations and the vast number of unique variants identified through WES (about 20–90,000 variants per individual) and WGS (about 3–5 million), the process of variant interpretation has become the bottleneck of molecular diagnostics.^50^

To facilitate and harmonize this process, bioinformatic pipelines (a series of computational steps) are implemented, and a handful of published guidelines and standards are followed in many countries and diagnostic laboratories.^51^, ^52^, ^53^

To summarize, variants are assessed according to a range of criteria such as the frequency of the variant in affected and unaffected individuals, the segregation of the variant within families, and computer‐generated assessments of evolutionary conservation and the severity of the change that may be caused by the variant. Physicians ordering genetic testing do not need to be able to apply these criteria but should understand that there are a range of factors used to estimate the probability of pathogenicity.^54^ If the probability that a variant is pathogenic is less than 10% it is termed *benign* (ie, without health consequences), 10% or greater and less than 90% is termed *variant of unknown significance* (VUS), between 90% and 99% is termed *likely pathogenic*, and greater than 99% is termed *pathogenic*.^54^ It is important to note that pathogenicity is a probabilistic assertion of the likelihood that the variant is causally related to a heritable disease—it is not a clinical diagnosis.

Unfortunately, there are challenges related to the variant interpretation guidelines. Firsty, it is stated that multiple variant pathogenicity prediction models should agree, and if they do not, prediction models should not be used at all for the assessment of that variant—as such, models with the poorest performance have the largest impact.^50^ Second, lack of bioinformatics expertise in clinical and research laboratories results in implementation of suboptimal methods, including outdated and overly similar tools leading to poorer and biased outcomes.^50^ To be explicit, bioinformatic tools for variant interpretation are often chosen based on their being mentioned in popular guidelines.^50^ However, benchmark studies comparing the prediction of state‐of‐the‐art methods with others indicate 30% or even larger differences, and thus popularity does not equal performance.^50^, ^55^, ^56^, ^57^ Third, there is a misconception that variants ought to be divided into binary, mutually exclusive categories of benign and pathogenic, whereas there are variations that cannot be placed in either category (VUS).^50^ These variants are particularly challenging, as at the time of classification, there is insufficient or conflicting evidence regarding the role of the molecular alteration in causing disease. The challenge of filtering VUS and making a diagnostic decision as well as future directions to unravel their role in disease biology are detailed later in the review.

### Perspective 3: Implementation of Current and Complementary Bioinformatic Tools

Much like other diagnostic techniques such as neuroimaging, bioinformatic tools continue to evolve and diagnostic laboratories need to be configured to update their pipelines as and when appropriate. Bioinformatic tools implemented should represent state‐of‐the‐art performance and should be complementary, not based on the same principles and reusing the same data and predictions.^58^ Moreover, the choice of the prediction methods should be based on the latest systematic benchmarking studies.^50^ Ideally, bioinformatic experts should publish consensus statements as and when important modifications ought to be implemented.

### Challenge 4: The Filtering of Variants Associated with Disease

Whereas variant interpretation is the process of assigning probabilistic labels, variant filtering is the pivotal step in which variants that are most likely to be clinically significant are shortlisted, enabling clinicians to arrive at a (clinical‐)molecular diagnosis.^59^ To be precise, the aim of variant filtering is to identify rare and protein‐changing variants in clinically relevant genes and differentiate deleterious mutations not related to the disease in question (variants of unknown significance and incidental findings).^59^

As variant classification is computationally intensive and requires cross‐referencing with prevalence data and prediction models, bioinformatic pipelines play a crucial role in variant filtering as well. However, despite their implementation, the prevailing challenge is that a single exome still contains about 100 to 200 potential disease‐causing changes.^59^

Consequently, an increasingly popular strategy has been to do panel testing using filtered WES and WGS data, that is, the exome or whole genome are sequenced but only the disease‐associated genes are analyzed.^60^ Advantages of this filtered approach include improved cost‐effectiveness, because the challenges of analyzing variants identified in clinically irrelevant or phenotypically unrelated genes is alleviated and a pathogenic mutation in a known disease‐causing gene can be identified more quickly, and the flexibility to expand the panel as needed if the initial analysis is negative or if new candidate genes are identified.^60^ However, the challenges of overlap in the clinical spectrum of different movement disorders and the subsequent tendency to filter genes incorrectly, confirmation bias, and a lack of consensus regarding what is a current and complete panel for a phenotype hinder variant filtering.^60^

Alongside computational approaches, functional testing can also be used to assess strongly filtered variants (new variants in known genes or genetic defects in potentially new disease‐causing genes). Although functional tests provide more insight into the biological implication of a defect, a lack of high‐throughput testing makes effective gene prioritization all the more crucial.^61^

### Perspective 4: A Clinical‐Molecular Diagnosis Through Close Collaboration Between Clinicians and Genetic Laboratories

When filtering variants associated with disease, the clinician plays a central role in making a clinical‐molecular diagnosis: Does this particular finding explain the symptoms this particular patient is presenting with?^62^ Similar to any test result, the identification of a genomic variant provides evidence for or against conditions that are (or should be) in the differential diagnosis. As such, the ordering clinician should integrate the genetic test result with the clinical characteristics and family history of the patient to arrive at a clinical‐molecular diagnosis. Whereas pathogenic variants are the easiest variants to interpret, the majority of variants are not highly predictive of the phenotype—specifically, variants of unknown significance or likely pathogenic mutations in known disease‐causing genes—and yet, a diagnostic decision still needs to be made.^54^ To this end, there is a need to train clinicians with a bridging role between clinical practice and genetic laboratories to enable close collaboration in multidisciplinary settings as well as effective genotype–phenotype integration. Michaelson‐Cohen and colleagues^63^ recently demonstrated the feasibility of improving genetics knowledge among all physicians from all backgrounds, years of practice, medicine disciplines, and positions.

### Challenge 5: The Lack of Standardized, Complete, and Up‐to‐Date Gene Lists

To achieve best‐in‐class diagnostic yield, it is important to use a current and complete disease‐causing gene list. Keeping gene lists up to date is time‐consuming, and curation is currently conducted through a manual literature search in an ad‐hoc manner by various research groups. Moreover, with the number of genes implicated in movement disorder pathophysiology continuing to increase, there has been a diffusion of differing multigene diagnostic panels for the various phenotypes, and it is unclear which of these gene lists to use.^64^

Dystonia is a striking example of this. Dystonia may appear isolated or in combination with other movement disorder phenotypes.^7^ There are currently 5 genes—*TOR1A*, *THAP1*, *GNAL*, *ANO3*, and *COL6A3*—that have been confirmed by 2 independent research groups as disease‐causing for the specific isolated presentation of dystonia. In addition to these 5 genes, many (more than 100 and counting) have been implicated in the genetic architecture of dystonia as several genetic disorders manifest with combined dystonia or with dystonia associated with other neurological or systemic features.^65^ As such, selecting a current and complete list of dystonia genes for diagnostic testing poses a significant challenge.

To illustrate the extent of this challenge, we searched PubMed for review articles with overviews of implicated genes published in the past 5 years using the search terms “dystonia AND genetic* AND movement disorder” and included isolated and combined dystonia and dystonia with associated features, with dystonia presenting as a prominent phenotype. The search yielded 180 results; we screened 180 titles and abstracts and reviewed 20 full articles. We included 13 articles with overviews of genes implicated in the genetic architecture of dystonia (Supplementary Table S1) and identified 203 unique genes. No 2 dystonia gene lists were the same, and we found considerable differences reported by various authors during the past 5 years as illustrated by Table 2.

**TABLE 2:** *Overview of the number of times genes were reported to be implicated in the genetic architecture of dystonia in the past 5 years (based on an analysis of 13 included articles)*

Number of Times Reported (Based on an Analysis of 13 Included Articles)	Overview of Genes Reported to be Implicated in the Genetic Architecture of Dystonia in the Past 5 Years
12	*THAP1*, *TOR1A*
11	*ANO3*, *GCH1*, *GNAL*, *SGCE*
10	*SPR*
9	*ATP1A3*, *PRKRA*, *TH*
8	*PLA2G6*, *TAF1*
7	*ATP7B*, *PANK2*, *SLC2A1*, *SLC30A10*, *WDR45*
6	*C19orf12*, *FA2H*, *TIMM8A*, *TUBB4A*
5	*ADCY5, ATP13A2, CP, DCAF17, GCDH, KMT2B, NPC1, NPC2, SLC19A3, SLC39A14, SLC6A3, SUCLA2*
4	*DDC, FTL, GLB1, PNKD, PRRT2, PTS*
3	*CIZ1, DNAJC12, FOXG1, GAMT, HPCA, KCTD17, MECP2, MUT, PCCA, PCCB, PDGFB, PDHA1, PLP1, PRKN, QDPR, SERAC1, SLC20A2, SURF1, TREX1, VPS13A, XPR1*
2	*ACAT1, ADAR1, ALDH5A1, ARX, ATM, BCS1L, CBS, COL6A3, COX10, COX15, DJ1, FOLR1, GM2A, HEXA, HPRT, HPRT1, HTT, MMAA, MMAB, mt‐ND6, NDUFA10, NDUFA12, NDUFA2, NDUFA9, NDUFAF2, NDUFAF6, NDUFS1, NDUFS3, NDUFS4, NDUFS7, NDUFS8, NKX2‐1, PAH, PARK2, PDGFRB, PDHX, PINK1, POLG, RNASEH2A, RNASEH2B, RNASEH2C, SAMHD1, SLC6A19, SLC6A8, SPG7, TPP1, TTPA*
1	*AADC, AARS, ABCD1, ADAR, ADCK3, AFG3L2, AGAT, APT1A3, APTX, ARSA, ATN1, ATXN3, BCAP31, BCKDHA, BCKDHB, C10orf2, C20orf7, C8orf38, CACNA1B, CASK, CDKL5, CLN3, CLN5, CLN6, CLN8, COASY, COQ8, COX20, CTSD, CTSF, CYP27A, D2HGHD, DBT, DDP, DLAT, DLD, DNAJC5, ECHS1, FBXO7, FLT, FOXRED1, FUCA1, FUS, GALC, GALT, GATM, GCH2, GJC2, GNAK, GNAO1, GNB1, GRN, HEXB, IPPK, JPH3, KCTD7, KIF1C, KMTB, LRPPRC, MCEE, MFSD8, MMADHC, MMUT, MOCS1, MR1, mt‐ATP6, MTFMT, MTTP, NDUF, NDUFAF5, NUP62, PANK, PCBD1, PDGRB, PDHB, PNKP, POLG1, PPT1, PRRT, RAB39B, RELN, SCN8A, SCO2, SDHA, SETX, SLC16A2, SLC20A1, SLCA19, SPG11, SQSTM1, SUCLG1, TACO1, TITF1, TUBB4, UBA5, XK*

See Supplementary Table S1 for a further specification of the genes reported per article.

### Perspective 5: Ongoing Curation of Gene Lists and Periodic Reanalysis of Genetic Sequencing Data

A number of databases where clinicians can share information on patients or families with rare or novel phenotypes and possibly disease‐causing variants to be able to refute or confirm genetic findings (eg, https://decipher.sanger.ac.uk/; https://phenomecentral.org; https://genematcher.org/; https://www.matchmakerexchange.org/) have been created. Although these facilitate the confirmation of candidate genes, it is important to shift toward a setting where there is 1 central repository moderated by neurogeneticists with domain‐specific expertise, and diagnostic laboratories globally are equipped with the same list of curated panel genes per phenotype.^21^ Such a central repository would require an interface where clinicians can annotate their cases with movement disorder–specific phenotype information such as Enroll‐HD and the European Friedreich's Ataxia Consortium for Translational Studies (EFACTS).^66^, ^67^ Movement Disorder Society taskforces could play an instrumental role in ensuring completeness and adherence to quality standards.

Being equipped with up‐to‐date gene lists per phenotype will also help us transition away from diagnostic genetic testing being a one‐off procedure, with the reanalysis of unsolved cases occurring on an ad‐hoc basis at the ordering physician's request. Dynamic and periodic reanalyses of genetic data are of paramount importance and are already yielding more molecular diagnoses.^68^, ^69^ In addition to the technical challenge of performing periodic reanalysis, the challenge of recontacting clinicians and patients also needs to be overcome.^70^, ^71^

## Future Directions to Tackle the One‐Phenotype‐Many Genes Paradigm

### Clinical Setting: Post‐NGS Phenotyping and a Shift Toward WGS

Post‐NGS phenotyping is a process—which was first proposed by Hennekam and Biesecker in 2012^72^ and has yet to be structurally implemented—in which NGS is performed before other clinical investigations. Hereafter, clinicians use their diagnostic skills to distinguish candidate mutations, revealed by NGS test results, through focused evaluations of their manifestations, that is, posttest diagnostic assessment mode as opposed to pretest differential diagnosis generation mode.^72^ With the rapidly decreasing costs of NGS, and first‐tier genetic testing using WGS showing improved diagnostic yield compared with standard clinical genetic testing and targeted resequencing panels, post‐NGS phenotyping using WGS is on the verge of becoming a reality.^73^

The advantages of such an approach include the following: (1) assessment of nucleotide sequence across the whole genome (eg, intronic and promoter regions) as well as copy number variants and many tandem repeat expansions through bioinformatic analyses, (2) more timely and comprehensive elucidation of disease‐causing mutations, (3) determination of new disease‐causing genes and new phenotypes associated with known genes, and (4) periodic reanalysis in case there is no initial diagnosis. Nonetheless, for successful implementation of post‐NGS phenotyping, the importance of addressing existing challenges is underscored. First, it is all the more important for the clinician to effectively rule out acquired causes and select other testing methods where appropriate (ie, mtDNA analysis, metabolic laboratory tests or neuroimaging). Second, utmost care ought to be taken to differentiate deleterious mutations not related to the disease in question (especially incidental findings), and ultimately, clinicians and genetic laboratories ought to collaborate in multidisciplinary settings to make clinical‐molecular diagnoses. We encourage institutions to share their experiences with the broader community so we learn how this compares to current practices and where there are opportunities for structural improvement.

### Research Setting: Cohort Analyses Enriched by Genotype–Phenotype Integration and Gene Networks

Particularly in rare disease research, often there is only 1 patient or family described with a possible mutation in a gene that has not yet been linked to the given disease.^59^, ^74^ For this reason, cohort analyses, enabled by data sharing and enriched by the integration of genotype data and deep phenotype information, are an invaluable way to discover new disease‐causing genes and establish the full clinical spectrum of movement disorders.^75^ To be specific, one could aggregate individuals with a highly comparable phenotype and evaluate genotype correlations, or vice versa, adjudge and adjust existing genotype–phenotype associations. Moreover, as it remains unclear whether the one‐phenotype‐many‐genes paradigm is due to a common phenotypic outcome of diverse underlying mechanisms or whether the different genetic causes converge in a single molecular pathway, gene networks are a promising approach to consider incorporating as well.^76^

**FIG. 1:**
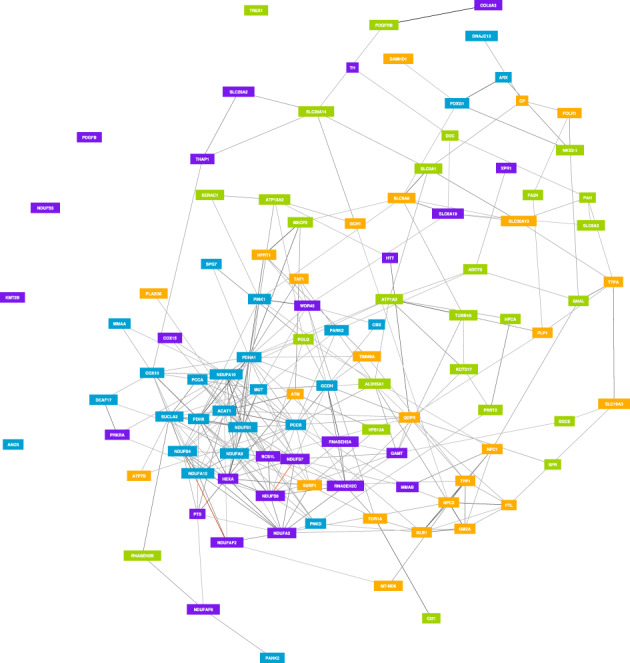
Gene network visualization of genes that have been reported with dystonia presenting as a prominent feature by 2 or more authors in the past 5 years (https://www.genenetwork.nl/). Genes likely to be involved in similar biological processes share a color and constitute a cluster. Connectedness between genes is depicted using gray lines. See Table 2 for an overview of the genes used to construct this network.

Gene networks are models used to construct and visualize gene–gene interactions based on the statistical analysis of gene expression (RNA sequencing) big data.^77^ The underlying hypothesis is that genes showing similar expression patterns, that is, “coexpressed” or “coregulated” genes, likely act on similar molecular pathways and have similar biological functions.^78^ Analytical methods include clustering to reveal subgroups of genes and functional annotation to increase understanding of their possible biological function. Dystonia, a phenotype where the majority of patients have no molecular diagnosis despite there having been more than 100 genes implicated, is an illustrative example that could benefit from a gene network approach.^79^ Using genes that have been reported with dystonia as a prominent feature by 2 or more authors in the past 5 years (Table 2) as a starting point, we constructed a dystonia‐specific gene network (Fig. 1). The rationale is that genes that are tightly regulated with known dystonia genes likely are involved in dystonia networks as well and are potential new candidate genes.^80^

The gene network reveals that the genes implicated in the genetic architecture of dystonia are likely involved in distinct molecular pathways. This is suggested by the presence of multiple subsets of dystonia genes (clusters) that have different gene expression profiles. Pathway enrichment analysis (Table 3) of the dystonia genes and subsets helps provide insight into dystonia pathophysiology based on statistical grounds alone. Whereas aggregate analysis of all genes shows metabolic and signal transduction pathways to be significant, analysis of the individual clusters and their corresponding phenotype could help reveal whether dystonia is indeed caused by diverse underlying mechanisms or whether they all converge in a single pathway that leads to excessive muscle contraction.^64^

**TABLE 3:** *Pathway enrichment analysis of genes that have been reported with dystonia presenting as a prominent feature by 2 or more authors in the past 5 years as well as subsets of these dystonia genes that have been clustered by similar gene coregulation (https://www.genenetwork.nl/)*

	All Included Dystonia Genes	Subset of Dystonia Genes 1 (Blue Cluster)	Subset of Dystonia Genes 2 (Green Cluster)	Subset of Dystonia Genes 3 (Purple Cluster)	Subset of Dystonia Genes 4 (Orange Cluster)
1	Metabolism (*P* = 2.5 × 10^−18^)	Branched‐chain amino acid catabolism (*P* = 1.6 × 10^−11^)	Cell cycle checkpoints (*P* = 1.1 × 10^−5^)	Mitochondrial translation initiation (*P* = 5.3 × 10^−6^)	Metabolism (*P* = 7.1 × 10^−8^)
2	Signal transduction (*P* = 6.2 × 10^−16^)	Glyoxylate metabolism and glycine degradation (*P* = 4.8 × 10^−11^)	Recognition of DNA damage by proliferating cell nuclear antigen–containing replication complex (*P* = 2.8 × 10^−5^)	Mitochondrial translation elongation (*P* = 6.8 × 10^−6^)	Metabolism of vitamins and cofactors (*P* = 9.1 × 10^−8^)
3	Metabolism of vitamins and cofactors (*P* = 9.6 × 10^−14^)	Complex I biogenesis (*P* = 7.2 × 10^−11^)	Transport of vitamins, nucleosides, and related molecules (*P* = 3.0 × 10^−5^)	Mitochondrial translation termination (*P* = 9.4 × 10^−6^)	Transport of small molecules (*P* = 3.4 × 10^−6^)
4	G protein‐coupled receptor downstream signaling (*P* = 4.9 × 10^−11^)	Pyruvate metabolism and citric acid cycle (*P* = 7.6 × 10^−11^)	Generic transcription pathway (*P* = 4.8 × 10^−5^)	Mitochondrial translation (*P* = 1.0 × 10^−5^)	Plasma lipoprotein assembly, remodeling, and clearance (*P* = 4.7 × 10^−6^)

The Bonferroni‐corrected significance threshold is 5.2 × 10^−5^ (α = 0.05 and 960 molecular pathways are tested for enrichment per gene set). The smaller the *P* value, the more likely the set of genes is implicated in the molecular pathway.

In addition to contributing to a better understanding of disease biology, gene networks can be used to determine the shared molecular mechanisms between various movement disorders and provide novel targets for disease therapies as illustrated by Nibbeling and colleagues^81^ for the spinocerebellar ataxias and dystonia. Moreover, as they can be used to identify previously unknown disease gene associations and prioritize candidate genes, they are particularly useful in conjunction with cohort analyses and genotype–phenotype integration for higher diagnostic yield.

## Conclusion

In this review, we shed light on the “one‐phenotype‐many‐genes” paradigm: the difficulty of identifying the 1 disease‐causing gene among the many possible genetic defects that may be responsible for a certain movement disorder phenotype. We systematically discuss the main challenges related to this topic and the corresponding perspectives to address them. Future directions to advance the frontiers of our understanding of disease biology for more molecular diagnoses and targeted therapy are post‐NGS phenotyping and a shift toward WGS in the clinical setting, and cohort analyses enriched by genotype–phenotype integration and gene networks in the research setting.

## Author Roles

(1) Manuscript Preparation: A. Writing the First Draft, B. Review and Critique.

R.G.: 1A

S.v.d.V.: 1A

M.v.E.: 1B

T.J.d.K.: 1B

M.A.J.T.: 1B

## Disclosures

### Ethical Compliance Statement

We confirm that we have read the Journal's position on issues involved in ethical publication and affirm that this work is consistent with those guidelines. An institutional review board or ethics committee did not review this work. Informed patient consent was not necessary for this work.

### Funding Sources and Conflicts of Interest

The authors declare that there are no conflicts of interest relevant to this work.

### Financial Disclosures for the Previous 12 Months

Tom J. de Koning reports grants from the Metabolic Power Foundation, the Piet Poortman Foundation, and the North Sea Myoclonus Foundation. Marina A.J. Tijssen reports grants from the Netherlands Organization for Health Research and Development ZonMW Topsubsidie (91218013), the European Fund for Regional Development from the European Union (01492947), and the province of Friesland, Dystonia Medical Research Foundation, Stichting Wetenschapsfonds Dystonie Vereniging, Fonds Psychische Gezondheid, and Phelps Stichting and an unrestricted grant from Actelion and AOP Orphan Pharmaceuticals AG.

## Supporting information


**Table S1**. Overview of review articles published in the past 5 years with overviews of genes implicated in the genetic architecture of dystonia. PubMed search dated May 2020; search terms: “dystonia AND genetic* AND movement disorder.”
